# The Cost-Effectiveness of Supplemental Carnosine in Type 2 Diabetes

**DOI:** 10.3390/nu14010215

**Published:** 2022-01-04

**Authors:** Kirthi Menon, Barbora de Courten, Dianna J. Magliano, Zanfina Ademi, Danny Liew, Ella Zomer

**Affiliations:** 1 School of Public Health and Preventive Medicine, Monash University, Melbourne, VIC 3004, Australia; kirthi.aravindmenon@monash.edu (K.M.); zanfina.ademi@monash.edu (Z.A.); danny.liew@monash.edu (D.L.); 2 Department of Medicine, School of Clinical Sciences, Monash University, Melbourne, VIC 3168, Australia; barbora.decourten@monash.edu; 3 Baker Heart and Diabetes Institute, Melbourne, VIC 3004, Australia; dianna.magliano@baker.edu.au

**Keywords:** carnosine, type 2 diabetes, prevention, cost-effectiveness

## Abstract

In this paper, we assess the cost-effectiveness of 1 g daily of carnosine (an over the counter supplement) in addition to standard care for the management of type 2 diabetes and compare it to standard care alone. Dynamic multistate life table models were constructed in order to estimate both clinical outcomes and costs of Australians aged 18 years and above with and without type 2 diabetes over a ten-year period, 2020 to 2029. The dynamic nature of the model allowed for population change over time (migration and deaths) and accounted for the development of new cases of diabetes. The three health states were ‘Alive without type 2 diabetes’, ‘Alive with type 2 diabetes’ and ‘Dead’. Transition probabilities, costs, and utilities were obtained from published sources. The main outcome of interest was the incremental cost-effectiveness ratio (ICER) in terms of cost per year of life saved (YoLS) and cost per quality-adjusted life year (QALY) gained. Over the ten-year period, the addition of carnosine to standard care treatment resulted in ICERs (discounted) of AUD 34,836 per YoLS and AUD 43,270 per QALY gained. Assuming the commonly accepted willingness to pay threshold of AUD 50,000 per QALY gained, supplemental dietary carnosine may be a cost-effective treatment option for people with type 2 diabetes in Australia.

## 1. Introduction

Globally, the prevalence of diabetes is rising and represents an important cause of preventable morbidity and mortality. Untreated diabetes over time can lead to irreversible long-term macrovascular and microvascular complications. The worldwide prevalence of diabetes in 2021 was 10.5%, and is expected to increase to 12.2% by 2045 [1]. In Australia, the age-standardised prevalence of self-reported diabetes increased from 3.3% in 2001 to 4.4% in 2017–2018 [2]. Diabetes was associated with 1.2 million hospitalisations in Australia in 2017–2018 [2], the majority due to type 2 diabetes, which is largely preventable. Not surprisingly, the associated economic costs are significant and opportunities to improve management are abundant. Evidence suggests that 12% of global health expenditure is spent on diabetes [3].

Over the years, the management of type 2 diabetes has evolved to encompass a wider range of pharmaceutical options to improve glycaemic control. Metformin is often first-line therapy, and additional pharmacotherapy is added as required depending on glycaemic targets, co-morbidities, and patient preferences. Despite recent advancements in treatment options, adequate glycaemic control remains a challenge for many people. Furthermore, the adverse effects and costs of pharmacotherapies are limitations.

Carnosine is a naturally occurring dipeptide that consists of two amino acids, beta-alanine and L-histidine [4]. It is present in human skeletal muscle, heart, and brain tissues [4], and has been shown to improve cardiometabolic risk factors in both animal [5,6] and human studies [7,8,9]. In addition, it has been shown to have positive effects on cognition, depression, and anxiety [10], all common in diabetes. The ‘typical’ dietary intake of carnosine (meat and fish being the main sources) is at concentrations insufficient to exert a biological effect [11]; however, carnosine is available as a dietary supplement. Several human studies have demonstrated an improvement in glycaemic measures (blood glucose levels, HbA1c) [7,8] and cardiovascular risk factors [8] with carnosine supplementation. Our recent systematic review and meta-analysis showed a reduction in HbA1c levels comparable to oral medications used to treat type 2 diabetes [9]. Importantly, it is safe, with an excellent side effect profile. Furthermore, it is freely available and relatively inexpensive.

Carnosine is believed to exert its benefits through its anti-inflammatory, anti-glycating, anti-oxidative and chelating properties [4]. The various properties of carnosine make it a potential treatment alternative for the management of chronic diseases, as the underlying pathophysiology of many of these diseases involves more than one pathway (as an example, both inflammation and oxidative stress underlie obesity).

There are no known published studies on the cost-effectiveness of carnosine in improving glycaemic control. In the present study, we sought to explore the cost-effectiveness of dietary supplemental carnosine in patients with type 2 diabetes.

## 2. Materials and Methods

### 2.1. Model

**Figure 1:**
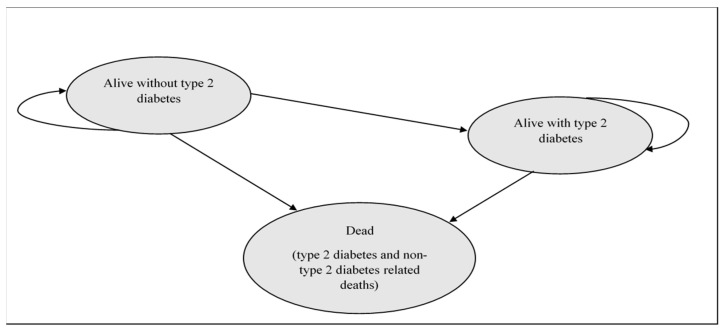
Illustration of the dynamic nature of the model which allows for movement between health states.

Our previously published dynamic model [12] was adapted to estimate years of life and quality adjusted life years (QALYs) lived by Australians aged ≥18 years over a ten-year period from 2020 to 2029. Healthcare costs incurred were also estimated. The model comprised three health states: ‘Alive, with no type 2 diabetes’, ‘Alive, with type 2 diabetes’ and ‘Dead’ (Figure 1). Decision analysis [13] was used to compare the effectiveness and costs of supplemental carnosine (in addition to standard care) versus standard care, targeted only towards people with type 2 diabetes. The dynamic nature of the model allowed for population change over time due to evolving mortality and migration trends. Additionally, it allowed for entry into the model of subjects aged 18 years and for individuals without type 2 diabetes to develop it during the model time horizon. The assumption was made that once an individual developed type 2 diabetes, remission was not possible.

### 2.2. Model Population

As an example of the dynamic nature of the model, in 2019 (the year before the baseline year of 2020), there were 147,520 males aged 50 years with no diabetes and 8561 with diabetes, determined from the Australian total population and diabetes prevalence data. In 2020, it was estimated that there would be 146,587 males aged 51 years with no type 2 diabetes (147,520 males with no diabetes in 2019 minus 360 people who died and 729 people who developed type 2 diabetes, plus 157 people who migrated to Australia in 2020 without type 2 diabetes). It was estimated that in this year there would also be 9212 males aged 51 years with type 2 diabetes (8561 males with type 2 diabetes in 2019 minus 89 deaths, plus 729 people who developed type 2 diabetes and 10 people who migrated to Australia in 2020 with type 2 diabetes). Movement of people through the model and over years was determined using the prevalence and incidence of type 2 diabetes, migration data and mortality risks described below.

The model was built using Microsoft Excel (Microsoft Corporation, Redmond, WA, USA), with the Risk Analysis Add-in for Microsoft Excel @RISK 8.1.1 Industrial Edition (Palisade) used to undertake the probabilistic sensitivity analysis (PSA).

The model population was profiled on the 2019 Australian population characteristics. This 2019 population represented the model population in the year before the baseline year of 2020. Age (stratified in five-year age bands) and sex-specific prevalence and incidence of type 2 diabetes were obtained from the National Diabetes Services Scheme (NDSS) for 2015, the year of the latest available data [14]. The NDSS is an Australian government initiative which subsidises diabetes management products and provides information to people with diabetes who are registered. To obtain values for each single year of age, prevalence and incidence were plotted against the midpoints of each five-year age group, and polynomial functions were fitted to obtain single age year prevalence and incidence (Supplementary materials S1 and S2). As one polynomial function did not fit the prevalence data accurately, the data were split to represent younger and older age cohorts of people with type 2 diabetes in order to better estimate prevalence by single year of age. Data were also split to better estimate incidence by single year of age. Prevalence data from the NDSS were reported in five-year age groups from 0 to >100 years. As the prevalence of type 2 diabetes was close to zero in those < 10 years for both males and females, the prevalence was assumed to be zero. Therefore, prevalence of type 2 diabetes for both males and females was calculated for 12–27 years and 28–100 years (where, for example, 12 reflects the midpoint of the 10–14 year age group). Incidence from the NDSS was reported for <20, 20–24, 25–29 years and continued for five-year age groups until 85–89 years for both males and females. For the age group < 20 years, 10 was the midpoint value used. Therefore, incidence of type 2 diabetes for both males and females were calculated from ages 10–32 and 32–87 years. The incidence for people aged 10 years was also applied to those aged < 10 years. Similarly, the incidence for people aged 87 years was applied to those aged 88 to 100 years. Polynomial functions were fitted for each age cohort to determine the incidence and prevalence by single year of age (using different formulas, as shown in Supplementary Materials S1 and S2, respectively). Migration data projections, stratified by single year age and sex, were obtained from the Australian Bureau of Statistics (ABS) population projections data for 2017 to 2066 [15]. Net migration was calculated from available immigration and emigration data using the medium assumption. As per the ABS data, the immigration and emigration estimates remained constant after 2027. Prevalence of type 2 diabetes by single year of age and sex for the Australian population (described above) was applied to the migration data projections to determine the number of new migrants with type 2 diabetes.

### 2.3. Mortality Risk

Age-group (in five-year age bands from 5–10 to 85+ years) and sex-specific mortality rates for people with type 2 diabetes were determined from the linkage of NDSS to the national death index for 2014 [14], the latest available year with complete data. To determine the mortality rates for people with type 2 diabetes by single year of age, the midpoint of each age group (assuming a midpoint value of 92 for people 85+ years) and respective values were plotted and exponential functions were fitted (Supplementary material S3). Mortality rates for people without type 2 diabetes were calculated using the mortality rates in the total population drawn from Australian population mortality data for the relevant year [16], as well as the prevalence of type 2 diabetes and mortality rates among people with type 2 diabetes. The formulae use to calculate mortality rates for people without type 2 diabetes are included in Supplementary material S3. We assumed that age-specific mortality remained constant throughout the model time horizon.

### 2.4. The Efficacy of Supplemental Carnosine in People with Type 2 Diabetes

A recently published meta-analysis (involving five studies and 365 participants) found a 0.76% (8.3 mmol/mol) reduction in HbA1c in the group randomised to carnosine.^9^ To estimate the clinical benefits of these changes, change in HbA1c was correlated with all-cause mortality, as per Stratton et al. [17], who estimated that every 1% (10.9 mmol/mol) reduction in HbA1c was associated with a 14% relative reduction in all-cause mortality. Therefore, treatment with carnosine was assumed, through its HbA1c lowering properties, to result in a 10.6% (0.76 × 14%) reduction in all-cause mortality.

### 2.5. Utilities

Utility values for the population without type 2 diabetes were assumed to be equivalent to utilities for the general Australian population derived from a publication by McCaffrey et al., which included 2908 individuals [18]. These values were age- (presented in age groups) and sex-specific. It was assumed that the utilities applied across the age groups. For subjects with type 2 diabetes, utility values were obtained from a publication by Zhang et al. which included 7327 individuals with type 2 diabetes [19]. These values were age-specific but not stratified by sex. The utility values for males and females were determined using data regarding (i) the overall utility values (for both males and females) in each age group, (ii) relative differences in values between males and females, as reported by Zhang et al., and (iii) the proportion of males in the study population, as reported by Zhang et al. (Supplementary material S4). The utilities for people with and without diabetes are included in Table S1.

### 2.6. Costs

Data on healthcare costs were sourced from a publication by Lee CM et al. in which the annual direct healthcare costs for a person with a normal glucose tolerance was estimated to be AUD 1446, while the costs for known type 2 diabetes (using the weighted average of individuals with and without complications of diabetes) was AUD 3135 (values for 2004/2005) [20]. The weighted average cost considers the costs for people with no complications, microvascular complications, macrovascular complications, and people with both micro- and macrovascular complications of diabetes. Using the Australian health price index [21], these values were equal to AUD 1932 and AUD 4190, respectively, in 2019/2020 values.

The cost of carnosine per day was derived from publicly available sources. A dose of 1 g/day was AUD 1.16/day (AUD 0.58 per 500 mg tablet) [22], equating to an annual cost of AUD 423.40.

### 2.7. Outcomes

Outcomes were years of life saved (YoLS), QALYs gained, healthcare costs, and incremental cost-effectiveness ratios (ICERs). These were determined as the difference in years of life lived, QALYs lived, and healthcare costs incurred for the Australian adult population with and without diabetes, assuming people with diabetes were treated with standard care compared to people with diabetes being treated with supplemental carnosine in addition to standard care.

As the model was dynamic, years of life lived for each year were calculated by the number of people in two consecutive years for a particular age divided by two. For example, there were 147,520 males aged 50 years without type 2 diabetes in 2019 and 150,815 in 2020. Therefore, there were 149,167 years of life lived for males aged 50 years without type 2 diabetes in 2020 [(150,815 + 147,520)/2]. The total years of life lived is therefore the sum of years of life lived for males and females with and without diabetes from the years 2020 to 2029.

QALYs were calculated by multiplying the years of life lived by the relevant utility scores, which were age, sex, and diabetes-specific. For example, the QALYs for males aged 50 years without type 2 diabetes was 134,251 (149,167 × 0.9, which represents the utility value for the general population aged 45–54 years).

Healthcare costs for people with and without diabetes were determined by multiplying years of life lived by the respective healthcare costs. For example, the healthcare costs for males aged 50 years without type 2 diabetes in 2020 was AUD 288,220,262 (149,167 × $1932, which represents the annual healthcare costs for people with normal glucose tolerance tests). Of note, the sum of the formulas presented may not add to the total value reported due to rounding of numbers. As with years of life lived, the total QALYs and total healthcare costs were the sum of QALYs and healthcare costs for males and females with and without diabetes from the years 2020 to 2029.

ICERs are used to measure the cost-effectiveness of an intervention relative to a comparator, calculated by dividing the difference in costs between the intervention and the comparator by the differences in years of life and QALYs lived. In Australia, a commonly-accepted arbitrary cost-effectiveness threshold is AUD 50,000 per QALY gained [23,24]. However, according to the World Health Organisation (WHO), the threshold to determine cost-effectiveness should be referenced against a country’s gross domestic product (GDP) per capita [25]. ICERs less than one time the GDP per capita are considered ‘high value’, while ICERs one to three times GDP per capita are considered ‘intermediate value’. The World Bank estimated that the GDP per capita in Australia in 2019 was AUD 77,348 (USD 54,907) [26].

A 5% discount rate per annum [27] was applied to years of life lived, QALYs lived and costs incurred beyond the first year of the time horizon. Therefore, the results reported represent the discounted values.

### 2.8. Scenario and Sensitivity Analyses

One-way sensitivity analyses were undertaken to test uncertainty surrounding key model input parameters. One-way sensitivity analyses applied upper and lower uncertainty bounds of: (i) utility values of individuals with and without type 2 diabetes; (ii) healthcare costs of individuals with and without type 2 diabetes; (iii) the cost of carnosine; (iv) the effect of carnosine on HbA1c; and (v) the relative reduction in mortality associated with HbA1c reductions. The lower and upper bounds for each input parameter are detailed in Table 1.

Additionally, a PSA was simultaneously undertaken to assess the uncertainty surrounding input parameters via a Monte Carlo simulation with 10,000 iterations. The parameter variation and distributions are included in Table 1. As per modelling recommendations by Briggs et al. [28], normal distributions were applied to the efficacy of carnosine and the reduction in all-cause mortality conferred by reductions in HbA1c, gamma distributions were applied to costs, and beta distributions were applied to utilities.

Lastly, a series of scenario analyses were performed to test certain model assumptions by (i) reducing the discount rate to 3% [27] and 0%; (ii) reducing the time horizon to five years; and (iii) assuming a 0.6% (6.6 mmol/mol) reduction in HbA1c from treatment with carnosine, as per Houjeghani et al. [8]. As the meta-analysis by Menon et al. included studies using other supplements in addition to carnosine in the intervention arm, this scenario analysis was undertaken to assess the effect of carnosine alone.

Additional analyses comparing carnosine to resveratrol and comparing resveratrol to standard care were also undertaken. Resveratrol is a plant-derived polyphenolic compound which has been shown to have beneficial effects on insulin sensitivity and glucose tolerance in animal studies [29,30]. However, systematic reviews on the therapeutic efficacy of resveratrol in human clinical studies have shown inconsistent results [31,32,33,34,35,36]. A systematic review and meta-analysis by Liu et al. demonstrated that consumption of resveratrol significantly reduced fasting glucose, insulin, HbA1c, and insulin resistance in people with type 2 diabetes [31]. Conversely, a meta-analysis by Zhu et al. demonstrated that resveratrol significantly reduced fasting glucose and insulin levels; however, the impact on HbA1c was negligible in people with type 2 diabetes [32]. Hausenblas et al. reported in their meta-analysis that for people with type 2 diabetes consumption of resveratrol significantly reduced HbA1c, although it did not reduce fasting glucose, insulin or insulin resistance [33]. The differences found by these meta-analyses are driven by varied inclusion criteria, as well as small sample sizes, short treatment/study duration, and the different dosages of resveratrol in the included studies. In terms of impact on HbA1c, Liu et al. included two studies and reported a mean reduction in HbA1c of 0.79% (95% CI 0.11 to 1.48%) [31], while Hausenblas et al. included four studies and reported a mean reduction in HbA1c of 0.43% (standard error 0.16) [33]. For our analyses, the therapeutic benefit of resveratrol was informed by Hausenblas et al. to provide a conservative estimate, which is in line with two other recent systematic reviews [34,35]. The studies included in this meta-analysis employed resveratrol dosages of 8 mg/day, 250 mg/day, 1 g/day and 3 g/day [33]. We therefore assumed the daily cost of resveratrol was AUD 2.43 (assuming a daily dose of 1 g/day and AUD 1.21 per 500 mg tablet, as per the median cost of the available brands of supplemental resveratrol) [22]. This equates to an annual cost of resveratrol of AUD 886.10.

## 3. Results

### 3.1. Base Case

In 2020, it was estimated that there were 1,191,859 Australians aged 18 years and above with type 2 diabetes. In the base case analysis (Table 2), the total number of years of life lived (discounted) in the standard care and carnosine groups over the ten-year period of 2020 to 2029 were 172,083,114 and 172,227,026, respectively, translating to a gain of 143,913 years of life lived with the addition of carnosine to standard care. Similarly, QALYs (discounted) were higher in the carnosine group compared to standard care, with a total of 115,862 QALYs gained as a result of treatment with carnosine. Net costs (discounted) were AUD 355,690,912,502 in the standard care group and AUD 360,704,195,577 in the carnosine group. These equated to ICERs of AUD 34,836 per YoLS and AUD 43,270 per QALY gained over the ten-year period. The ICERs were AUD 37,718 and AUD 52,478 per QALY gained for males and females, respectively. Results stratified by gender can be found in Table S2.

### 3.2. Scenario Analyses

Assuming a discount rate of 3.0% resulted in an ICER of AUD 42,165 per QALY gained; without discounting, an ICER of AUD 40,592 per QALY gained was observed. Reducing the time horizon to five years increased the ICER to AUD 72,764 per QALY gained. Assuming a 0.6% (6.6 mmol/mol) reduction in HbA1c, as per the study by Houjeghani et al. [8], resulted in an ICER of AUD 53,434 per QALY gained.

Our scenario analyses exploring the cost-effectiveness of resveratrol demonstrated that supplemental resveratrol in addition to standard care was not cost-effective compared to standard care alone (ICER of AUD 146,089 per QALY gained). A direct comparison of resveratrol to carnosine showed that carnosine was dominant over resveratrol (cost-saving).

### 3.3. Sensitivity Analyses

When the lower- and upper-bound utility values for the population with diabetes were applied, the ICERs were AUD 43,752 and AUD 42,796 per QALY gained, respectively. Applying the lower bound of healthcare costs for diabetes resulted in an ICER of AUD 42,124 per QALY gained, while applying the upper bound resulted in an ICER of AUD 44,412 per QALY gained. This was a consequence of carnosine increasing longevity in people with type 2 diabetes, and hence the costs of its treatment. When the cost of carnosine was reduced and increased by 50%, the ICERs were AUD 24,237 and AUD 62,302 per QALY gained, respectively. Applying the lower- and upper-bound values of the effect of carnosine on HbA1c reduction had the greatest impact on the model (AUD 125,858 and AUD 27,609, respectively). Lastly, the lower and upper bounds of the reduction in mortality associated with HbA1c reduction resulted in ICERs of AUD 64,446 and AUD 33,239 per QALY gained, respectively. The results of the scenario and sensitivity analyses are presented in Table 3.

**Figure 2:**
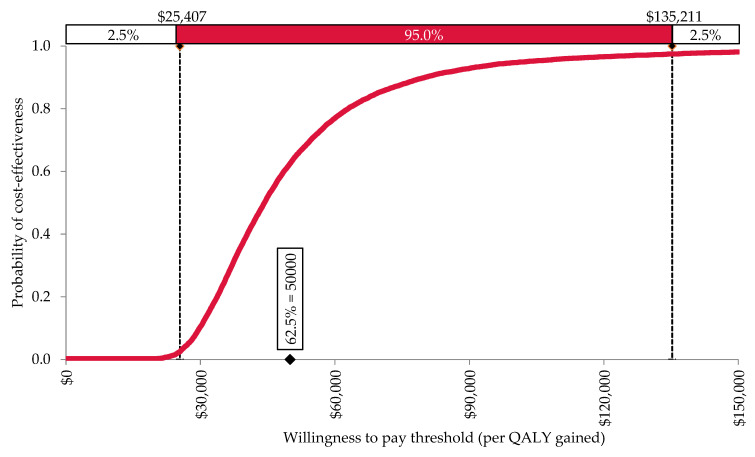
The cost-effectiveness acceptability curve, demonstrating that 62.5% of iterations were below Australia’s commonly-accepted willingness to pay threshold of AUD 50,000 per QALY gained. Results are reported in 2020 Australian dollars (AUD).

Assuming a willingness to pay threshold of AUD 50,000 per QALY gained, the PSA demonstrated that carnosine in addition to standard care was cost-effective when compared to standard care only in 62.5% of iterations. The 2.5th and 97.5th percentiles were AUD 25,407 and AUD 135,211, respectively (Figure 2).

## 4. Discussion

The findings from this study suggest that the addition of supplemental carnosine to standard care is likely to be a cost-effective treatment for the management of type 2 diabetes based on commonly-accepted willingness-to-pay thresholds for ICERs [23,24,25]. While the Pharmaceutical Benefits Advisory Committee (PBAC) in Australia does not have an explicit cost-effectiveness threshold, a medication with a cost less than AUD 50,000 per QALY gained is more likely to be recommended for public funding [37]. However, there is substantial variability in terms of what constitutes an acceptable ICER [24,38,39]. At least according to WHO guidelines [25], carnosine would be considered a ‘high value’ intervention, given the ICERs we estimate fall within Australia’s GDP per capita.

To our knowledge, there have been no other cost-effectiveness studies of carnosine on any health outcomes to date, nor any that have used a dynamic model approach. The effect of carnosine on HbA1c as demonstrated in the study by Menon et al. [9] is comparable to the effect of some of the other oral hypoglycaemic medications, including dipeptidyl peptidase IV inhibitors and alpha glucosidase inhibitors [40]. The efficacy of carnosine in combination with its favourable safety profile makes it an attractive option for patients with type 2 diabetes. In addition, our sub-analysis highlights that compared to standard care, carnosine is more cost-effective than resveratrol, another available supplement shown to have insulin sensitivity and glucose control benefits. In fact, carnosine is dominant in terms of cost savings compared to resveratrol. This is a result of the greater efficacy of carnosine on HbA1c reduction as well as its lower acquisition price. Supplements are not typically reimbursed in Australia, and are sourced and paid for out-of-pocket by the individual. Our study highlights that from the Australian health care perspective, carnosine is likely to be cost-effective, and therefore the Australian government should consider its reimbursement for people with type 2 diabetes.

Cost-effectiveness decreased when the time horizon was five years, which was expected; for long-term preventive treatments that are administered, and hence costed, on a regular basis, cost-effectiveness is typically highly sensitive to time horizon. Other input parameters to which the results were sensitive were the effect of carnosine on HbA1c, its cost, and the mortality benefits associated with reductions in HbA1c. Overall, the sensitivity analyses supported both the robustness of the model and our conclusion that carnosine is likely to be cost-effective.

There are several strengths of our study. First, our model included all Australian adults (aged ≥ 18 years), and its dynamic nature accounted for migration and death, providing a more accurate estimate of the evolving demographics of the Australian population over a ten-year period. The model also allowed for incident cases of type 2 diabetes to be captured during this time period. Additionally, the input measures, including the incidence and prevalence of type 2 diabetes and mortality data, were age- and sex-specific and derived from Australian population national data.

However, our study was not without limitations. The effect of carnosine on HbA1c obtained from a meta-analysis included studies that used carnosine together with other supplements in the intervention arm [9]. Additionally, one study was focused on paediatric patients with type 1 diabetes [41]. However, in the scenario where the treatment effects of carnosine alone were applied, carnosine remained likely to be cost-effective. All studies were short in duration (<4 months). Long-term studies on carnosine are currently lacking, although these are of the utmost importance. Another limitation in the study is that we only explored the relationship between HbA1c and all-cause mortality, and disregarded the relationship between a reduction in HbA1c and other clinical outcomes such as cardiovascular mortality, as well as quality of life. We did not include the impact of carnosine on people with pre-diabetes. Therefore, we may have underestimated both the benefits and cost-effectiveness of carnosine. Additionally, we did not consider the complications or comorbidities associated with diabetes explicitly in the model. However, the risk of death for people with type 2 diabetes was drawn from NDSS data which include people at various stages of diabetes, including those with complications and comorbidities. The NDSS is estimated to capture 80–90% of the Australian population with diagnosed diabetes [42]. However, as the NDSS subsidises diabetes management products to people who are registered, it is likely to be biased and represent a more severe group of people with diabetes. That is, it is likely to capture people with type 2 diabetes requiring treatment; however, it may not include people with diet-controlled type 2 diabetes who do not need the services provided. Furthermore, the healthcare costs for people with type 2 diabetes used in our model represented the weighted average value derived from costs for people with type 2 diabetes with no complications, microvascular complications, macrovascular complications and both microvascular and macrovascular complications. The utility values for the population with diabetes were obtained from a US study, which may not be representative of the Australian population. We assumed 100% adherence to carnosine, which is probably not an accurate reflection of reality. However, unless adherence to a preventive intervention is very low, its cost-effectiveness remains steady because of the proportional changes to any benefits and costs.

## 5. Conclusions

In summary, the addition of carnosine to standard care likely represents a cost-effective treatment option for the management of type 2 diabetes among Australian adults.

## Figures and Tables

**Table 1:** Input parameters for the model.

Parameter	Base Case	Lower Limit 95% CI	Upper Limit 95% CI	Distribution for PSA	Source
*Utilities*					
No type 2 diabetes	see Table S1			Beta	McCaffrey et al. [18]
With type 2 diabetes	see Table S1			Beta	Zhang et al. [19]
*Disease costs*					
No type 2 diabetes	$1932	$1795	$2071	Gamma	Lee et al. [20]
With type 2 diabetes	$4190	$3268	$5110	Gamma	Lee et al. [20]
*Treatment costs*					
Annual carnosine cost *	$423.40	$211.70	$635.10	Fixed	iHerb [22]
*Carnosine treatment effect*					
Reduction in HbA1c (%)	0.76	0.24	1.29	Normal	Menon et al. [9]
*HbA1c and all-cause mortality*					
Reduction in HbA1c and all-cause mortality (%)	14.00	9.00	19.00	Normal	Stratton et al. [17]

* Lower and upper bounds for the annual cost of carnosine reflect ± 50% of the mean cost respectively. PSA, probabilistic sensitivity analysis. All costs are reported in Australian dollars (AUD) for the year 2020.

**Table 2:** Base case results for the total Australian population aged >18 years, with type 2 diabetes treated with carnosine plus standard care, compared to standard care alone over ten years.

Parameter	Standard Care Only	Standard Care + Carnosine	Difference
*Clinical parameters*			
Total years of life lived	172,083,114	172,227,026	143,913
Total QALYs	155,139,783	155,255,645	115,862
*Costs parameters*			
Disease costs	$355,690,912,502	$356,293,906,561	$602,994,059
Treatment costs	$0	$4,410,289,017	$4,410,289,017
Total healthcare costs	$355,690,912,502	$360,704,195,577	$5,013,283,075
*Incremental cost-effectiveness ratios*			
Costs per YoLS			$34,836
Costs per QALY			$43,270

QALY, quality of life years; YoLS, years of life saved. All costs are reported in Australian dollars (AUD) for the year 2020.

**Table 3:** Scenario and sensitivity analysis.

	Base Case Values	Scenario/ Variation	Upper and Lower Bound Values	ICER	
				Cost per YoLS	Cost per QALY
Base Case *				$34,836	$43,270
*Scenario analysis*					
Discounting	5%	3%		$33,946	$42,165
		0%		$32,679	$40,592
Time horizon	10 years	5 years		$58,595	$72,764
Effect of carnosine on HbA1c	0.76% (8.3 mmol/mol)	0.6% (6.6 mmol/mol)		$43,019	$53,434
Resveratrol versus standard care				$117,612	$146,089
Carnosine versus resveratrol				Dominant	Dominant
*Sensitivity analysis*					
Utility (no diabetes population) ^†^	see Table S1	95% CI	see Table S1	$34,836	$43,270
				$34,836	$43,270
Utility (diabetes population)	see Table S1	95% CI	see Table S1	$34,836	$43,752
				$34,836	$42,796
Healthcare costs (no diabetes population) ^†^	$1932	95% CI	Lower bound: $1795	$34,836	$43,270
			Upper bound: $2071	$34,836	$43,270
Healthcare costs (diabetes population)	$4190	95% CI	Lower bound: $3268	$33,914	$42,124
			Upper bound: $5110	$35,756	$44,412
Carnosine cost	$423.40	±50%	Lower bound: $211.70	$19,513	$24,237
			Upper bound: $635.10	$50,158	$62,302
Effect of carnosine on HbA1c	0.76%	95% CI	Lower bound: 0.24%	$101,323	$125,858
			Upper bound: 1.29%	$22,228	$27,609
Mortality reduction for every 1% reduction in HbA1c	14%	95% CI	Lower bound: 9.00%	$51,884	$64,446
			Upper bound: 19.00%	$26,760	$33,239

* The base case assumed a time horizon of 10 years and a 5% discount rate. ^†^ As the model was developed to assess the costs and benefits of treating people with diabetes with carnosine, varying the utilities and healthcare costs in people without diabetes did not alter the results. QALY, quality of life years; YoLS, years of life saved. All costs are reported in Australian dollars (AUD) for the year 2020.

## Data Availability

All data generated or analysed during this study are included in this published article (and its Supplementary Information Files).

## Supplementary Materials

The following are available online at https://www.mdpi.com/article/10.3390/nu14010215/s1, Supplementary material S1: Polynomial functions used to determine the prevalence of type 2 diabetes and for (a) males and (b) females, Supplementary material S2: Polynomial functions used to determine the incidence of type 2 diabetes for (a) males and (b) females, Supplementary material S3: Exponential functions used to determine the mortality risks for (a) males and (b) females with type 2 diabetes, Supplementary material S4: (a) Age-group specific utility values for people with type 2 diabetes and (b) calculations used to determine utility values stratified by gender, Table S1: Utility values for the general population and for those with type 2 diabetes stratified by age-group and gender, Table S2: Base case results for the total Australian population aged ≥ 18 years, including people with type 2 diabetes treated with carnosine plus standard care compared to standard care alone, over ten years for (a) males and (b) females.
