# Supplementary material for: The Cost-Effectiveness of Supplemental Carnosine in Type 2 Diabetes
Source: Nutrients. 2022 Jan 4;14(1):215. doi: 10.3390/nu14010215 (PMC8747040; doi:10.3390/nu14010215)
Supplement: Supplementary file 1 [file nutrients-14-00215-s001.zip › nutrients-1431160-supplementary.pdf]

Supplementary Appendices

The cost-effectiveness of supplemental carnosine in type 2 diabetes

Kirthi Menon[1] Barbora de Courten[2], Dianna J Magliano[3], Zanfina Ademi[1], Danny Liew[1], Ella Zomer[1]

[1]School of Public Health and Preventive Medicine, Monash University, Melbourne, Australia

[2]Department of Medicine, School of Clinical Sciences, Monash University, Melbourne, Australia

[3]Baker Heart and Diabetes Institute, Melbourne, Australia

Supplementary Appendices

Supplementary material S1: Polynomial functions used to determine the prevalence of type 2 diabetes and for a) males and b) females.

Supplementary material S2: Polynomial functions used to determine the incidence of type 2 diabetes for a) males and b) females.

Supplementary material S3: Exponential functions used to determine the mortality risks for a) males and b) females with type 2 diabetes.

Supplementary material S4: a) Age-group specific utility values for people with type 2 diabetes and b) calculations used to determine utility values stratified by gender.

Table S1: Utility values for the general population and for those with type 2 diabetes stratified by age-group and gender.

Table S2: Base case results for the total Australian population aged ≥18 years, including people with type 2 diabetes treated with carnosine plus standard care compared to standard care alone, over ten years for a) males and b) females.

Supplementary material S1: Polynomial functions used to determine the prevalence of type 2 diabetes for a) males and b) females.

a) Males

Type 2 diabetes (aged 12-27 years)

$$y = 0.000000230616141x^3 - 0.000004597313685x^2 + 0.000029538768311x$$

$R^2 = 0.997$

N.B. for ages <12 years, prevalence was zero.

Type 2 diabetes (aged 28-100 years)

$$y = 0.000000000013219x^6 - 0.000000003830368x^5 + 0.000000378606485x^4 - 0.000015029462557x^3 + 0.000246561324246x^2 - 0.001174334679718x$$

$R^2 = 0.996$

b) Females

Type 2 diabetes (aged 12-27 years)

$$y = 0.000000272625984x^3 - 0.000004658870780x^2 + 0.000029639477987x$$

$R^2 = 0.998$

N.B. for ages <12 year, prevalence was zero.

Type 2 diabetes (aged 28-100 years)

$$y = 0.000000000016837x^6 - 0.000000005428267x^5 + 0.000000643650918x^4 - 0.000035239047080x^3 + 0.000933700492837x^2 - 0.009504027528124x$$

$R^2 = 0.997$

where x denotes year of age; y denotes the estimated prevalence

Of note, as one polynomial function did not fit the prevalence data accurately, the data was split to represent younger and older age cohorts of people with type 2 diabetes to better estimate prevalence by single year of age. Prevalence from the NDSS were reported in five-year age groups from 0 to >100 years. As the prevalence of type 2 diabetes was close to zero in those <10 years for both males and females, the prevalence was assumed to be zero. Therefore, prevalence of type 2 diabetes for both males and females were calculated for 12-27 years and 28-100 years.

Supplementary material S2: Polynomial functions used to determine the incidence of type 2 diabetes for a) males and b) females.

a) Males

Type 2 diabetes (aged 10-31 years)

$y = 0.000032507459442x^3 - 0.001688215437892x^2 + 0.028897911959595x - 0.150834358583757$

$R^2 = 1.000$

N.B. for ages <12 year, incidence remained the same as 10 years old.

Type 2 diabetes (aged 32-100 years)

$y = 0.000000000486043x^6 - 0.000000136846820x^5 + 0.000014780691461x^4 - 0.000772575726245x^3 + 0.019993461489776x^2 - 0.202529037116619x$

$R^2 = 0.996$

b) Females

Type 2 diabetes (aged 10-31 years)

$y = 0.000014077224316x^3 - 0.000655140960758x^2 + 0.010878949971964x - 0.053528246349662$

$R^2 = 1.000$

N.B. for ages <12 year, incidence remained the same as 10 years old.

Type 2 diabetes (aged 32-100 years)

$y = 0.000000000076070x^6 - 0.000000017564259x^5 + 0.000001305949275x^4 - 0.000031422997722x^3 + 0.000008003848961x^2 + 0.007668471549550x$

$R^2 = 0.996$

where x denotes year of age; y denotes the estimated incidence

Of note, as one polynomial function did not fit the incidence data accurately, the data was split to represent younger and older age cohorts of people with type 2 diabetes to better estimate incidence by single year of age. Incidence from the NDSS were reported as <20, 20-24, 25-29 years and continued for five-year age-groups until 85-89 years for both males and females. For the age-group <20 years, 10 was the midpoint value used. Therefore, incidence of type 2 diabetes for both males and females were calculated from ages 10-32 and 32-87 years. The incidence for people aged 10 years was also applied to those aged <10 years. Similarly, the incidence for people aged 87 years was applied to those aged 88 to 100 years.

Supplementary material S3: Exponential functions used to determine the mortality rates for a) males and b) females with type 2 diabetes.

a) Males

$y = 0.59586579e^{0.05932771x}$

$R^2 = 0.967$

b) Females

$y = 0.23311414e^{0.06646266x}$

$R^2 = 0.918$

where x denotes year of age; y denotes the estimated mortality rate

Mortality rates for people without diabetes were calculated using the following equation

$$Mort_{no\ diabetes} = (Mort_{total} - Mort_{diabetes} * prevalence_{diabetes})/(1 - prevalence_{diabetes})$$

where $Mort_{no\ diabetes}$ denotes the mortality rates for people without diabetes; $Mort_{total}$ denotes the mortality rates in the total Australian population; $Mort_{diabetes}$ denotes the mortality for people with diabetes estimated using the exponential functions provided above; and $prevalence_{diabetes}$ is the prevalence of diabetes for that year (estimated from the number of people in that year with type 2 diabetes divided by the total population in that year)

Supplementary material S4: a) Age-group specific utility values for people with type 2 diabetes and b) calculations used to determine utility values stratified by gender.

a)

	**Total number (n)**	**EQ-5D for people with type 2 diabetes (mean, SD)**
**Age-group (years)**		
<35	106	0.82 (0.18)
35-44	580	0.81 (0.19)
45-54	1576	0.79 (0.19)
55-64	1971	0.80 (0.19)
≥65	3094	0.80 (0.17)
**Gender**		
Males	3432	0.82 (0.17)
Females	3895	0.78 (0.19)
Ratio males: females	3432/(3432+3895)=0.468	0.82/0.78=1.05

b) Sample calculation to determine the EQ-5D for males and females separately.

Assuming the EQ-5D for people with diabetes aged 35-44 years is 0.81, the following formulae were employed:

To calculate EQ-5D for females aged 35-44 years:

$EQ\text{-}5D_{females} = EQ\text{-}5D_{total} \div [(prevalence_{males} * \text{risk ratio of EQ-5D}) + (1 - prevalence_{males})]$

Therefore, 0.81/(0.468*1.05) + (1-0.468) = 0.79

To calculate EQ-5D for males aged 35-44 years:

$EQ\text{-}5D_{males} = EQ\text{-}5D_{females} * \text{risk ratio of EQ-5D}$

Therefore, 0.79*1.05 = 0.83

where $EQ\text{-}5D_{total}$ is the EQ-5D score in the total population (including both males and females); $prevalence_{males}$ is the prevalence of males in the total population; the risk ratio of EQ-5D is the ratio of EQ-5D scores in males compared to females.

Table S1: Utility values for the general population and for those with type 2 diabetes stratified by age-group and gender.

Age-group (years)	General population (mean)*		General population (lower bound)		General population (upper bound)		Diabetes (mean)		Diabetes (lower bound)		Diabetes (upper bound)	
	**M**	**F**	**M**	**F**	**M**	**F**	**M**	**F**	**M**	**F**	**M**	**F**
0-24	0.96	0.95	0.95	0.94	0.97	0.96	0.84	0.80	0.79	0.75	0.89	0.85
25-34	0.95	0.95	0.94	0.94	0.96	0.96	0.84	0.80	0.79	0.75	0.89	0.85
35-44	0.93	0.91	0.91	0.89	0.95	0.93	0.83	0.79	0.81	0.77	0.85	0.81
45-54	0.90	0.87	0.88	0.85	0.92	0.89	0.81	0.77	0.80	0.76	0.82	0.78
55-64	0.90	0.88	0.88	0.86	0.92	0.90	0.82	0.78	0.81	0.77	0.83	0.79
65-74	0.87	0.87	0.84	0.85	0.90	0.89	0.82	0.78	0.81	0.77	0.83	0.79
75-100	0.85	0.82	0.82	0.79	0.88	0.85	0.82	0.78	0.81	0.77	0.83	0.79

M: males, F: females

Utility scores for the general population were drawn from a publication by *McCaffrey et al* and applied to the health state 'alive without type 2 diabetes'.

The utility values remained constant across age-groups from which the data were drawn.

*Utility values for the general population were applied to the population with no diabetes.

Table S2: Base case results for the total Australian population aged ≥18 years, including people with type 2 diabetes treated with carnosine plus standard care compared to standard care alone, over ten years for a) males and b) females.

a) Males

Parameter	Standard care only	Standard care + carnosine	Difference
Clinical benefits			
Total years of life lived	84,413,274	84,501,306	88,032
Total QALYs	76,878,098	76,950,381	72,283
Costs			
Disease Costs	$175,475,317,234	$175,844,169,902	$368,852,669
Treatment Costs	$0	$2,357,474,879	$2,357,474,879
Total healthcare costs	$175,475,317,234	$178,201,644,781	$2,726,327,547
Incremental cost effectiveness ratios			
Costs per YoLS			$30,970
Costs per QALY			$37,718

b) Females

Parameter	Standard care only	Standard care + carnosine	Difference
Clinical benefits			
Total years of life lived	87,669,839	87,725,720	55,881
Total QALYs	78,261,685	78,305,264	43,579
Costs			
Disease Costs	$180,215,595,269	$180,449,736,658	$234,141,390
Treatment Costs	$0	$2,052,814,138	$2,052,814,138
Total healthcare costs	$180,215,595,269	$182,502,550,796	$2,286,955,528
Incremental cost effectiveness ratios			
Costs per YoLS			$40,925
Costs per QALY			$52,478

QALY: quality adjusted life years, YoLS: year of life saved

All costs are reported in 2020 Australian dollars (AUD).
